# Methane emissions in growing heifers while eating from a feed bin compared with 24-hour emissions and relationship with feeding behavior

**DOI:** 10.3168/jdsc.2021-0184

**Published:** 2022-05-05

**Authors:** Ashraf Biswas, Ajmal Khan, Dongwen Luo, Arjan Jonker

**Affiliations:** 1 AgResearch Limited, Grasslands Research Centre, Palmerston North 4410, New Zealand; 2 Department of Animal Science and Nutrition, Chattogram Veterinary and Animal Science University, Khulshi-4225, Chattogram, Bangladesh

## Abstract

The objective of the current study was to determine the relationship of daily CH_4_ emissions estimated during mealtime compared with measured daily CH_4_ emissions, and determine the relationship with feeding behavior, in growing heifers fed alfalfa silage in respiration chambers. Data from 8 growing cattle (Hereford × Holstein-Friesian) individually housed in 4 respiration chambers and fed ad libitum alfalfa silage delivered in Insentec feed-bins to record feeding behavior and intake were used. The 4 chambers are linked to 1 analyzer, which measures CH_4_ in each chamber approximately every 3 min. Each 3-min measurement was expressed as grams per day and averaged per 24 h or per time during a meal. A strong correlation (r = 0.88; determined using Deming regression) was observed between CH_4_ emissions (g/d) during mealtime (276 ± 22.7 g/d) and measured over 24 h (262 ± 24.0 g/d), without apparent systematic bias. Feeding behavior parameters that were correlated with CH_4_ yield (g/kg dry matter intake) in the current study were a negative correlation with the number of visits to the feed bin (r = −0.45), average meal size (r = −0.57), and average daily eating rate (r = −0.48). In summary, CH_4_ measured during meals was similar to 24-h measured CH_4_ output in growing heifers fed ad libitum alfalfa silage in respiration chambers, and some feeding behavior parameters, based on feed bin visits, explained some of the variation in CH_4_ yield between animals.

## Highlights

• Methane emissions during mealtime, converted to daily emissions, were compared with 24-h methane emissions from heifers fed ad libitum alfalfa silage in respiration chambers.
• Methane measured during meals was similar to 24-h measured CH_4_ output.
• Visits to the feed bin, average meal size, and average daily eating rate were natively related with CH_4_ per unit of intake.

## Graphical Abstract

**Figure:**
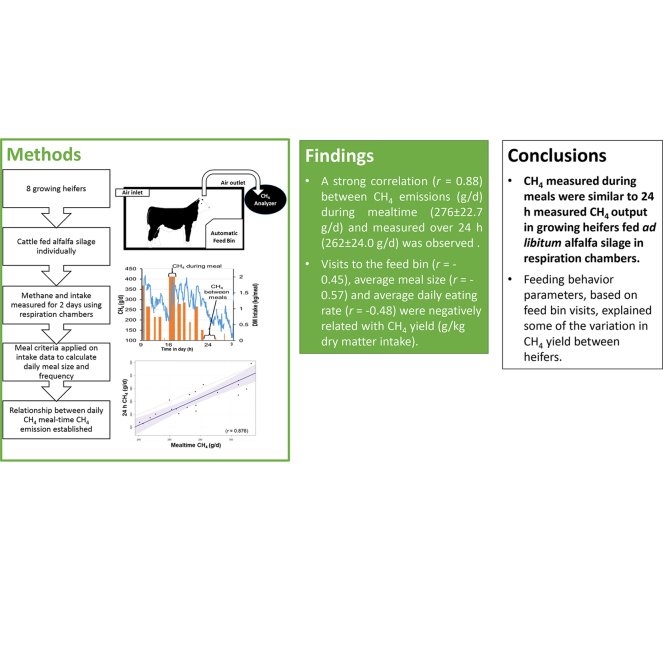
**Summary:** We determined the relationships between daily CH4 emissions estimated during mealtime and measured daily CH_4_ emissions, and that with feeding behavior in growing heifers (Hereford × Holstein-Friesian; n = 8) fed alfalfa silage in respiration chambers, which were linked to an analyzer that measured CH_4_ in each chamber every 3 min. Each 3-min measurement was expressed as grams per day and averaged per 24 h or per time during a meal. We observed a strong correlation (r = 0.88) between CH_4_ emissions (g/d) during mealtime and measured over 24 h, without apparent systematic bias. Feeding behavior parameters correlated with CH_4_ yield were negatively correlated with the number of visits to the feed bin (r = −0.45), average meal size (r = −0.57), and average daily eating rate (r = −0.48). In summary, CH_4_ measured during meals was similar to 24-h measured CH_4_ output in growing heifers fed ad libitum alfalfa silage in respiration chambers. Some feeding behavior parameters explained some of the variation in CH_4_ yield between animals.

Methane emitted by ruminants is a potent greenhouse gas (**GHG**) that constitutes approximately 15% of global GHG emissions (Gerber et al., 2013) and approximately 33% of total GHG emissions in New Zealand (MfE, 2017). Therefore, a large body of research is in progress to find mitigation options, which has sparked the development of cheaper and more practical methods with higher throughput to measure CH_4_ from ruminants. Many of these new methods estimate emissions based on multiple short-term (spot-samples) measurements from individual ruminant animals. One spot-sampling strategy developed is based on analyzing breath samples while the animal is visiting a feed bin with forage or TMR (Troy et al., 2016; Flay, 2018). However, the rate of CH_4_ emissions is not constant during the day and is affected by diet, feed allowance, and feeding pattern (Müller et al., 1980; Jonker et al., 2014), which might affect the predictive power of CH_4_ spot-sampling methods. Currently, little is known about the accuracy of methane emission estimates based on breath sample analysis during feeding.

The objective of the current study was to determine the relationship of daily CH_4_ emissions estimated during mealtimes compared with measured daily CH_4_ emissions and the relationship with feeding behavior in growing heifers fed alfalfa silage in respiration chambers. The hypothesis was that there would be a strong relationship between daily CH_4_ emissions estimated during mealtimes compared with measured daily CH_4_ emissions.

The animal experiment reported here was reviewed and approved by the AgResearch Grasslands Animal Ethics Committee (Palmerston North, New Zealand), and heifers were cared for according to the AgResearch Code of Ethical Conduct (AgResearch CEC, 2018). Data from 8 growing heifers (Hereford × Holstein-Friesian; BW = 487 ± 29 kg) were used for the current analysis (Jonker et al., 2014, 2016). Animals were fed ad libitum alfalfa silage, which contained 369 g/kg DM of NDF, 238 g/kg DM of CP, and 10.4 MJ/kg DM of ME calculated according to NRC (2000). During the measurement period, the animals were individually housed in 4 respiration chambers for 3 (first group of 4 over the weekend) or 2 (second group of 4 during the week) consecutive days. The silage was fed in Insentec feed-bins on loadcells (Hokofarm Group BV) inside the chambers with bins refilled at approximately 0800 and 1530 h. Airflow rate was 1.8 m^3^/min in each chamber and therefore the time required to exchange the chamber air was approximately 9 min. The 4 chambers were linked to a switching unit that directs the air stream of each chamber to one gas analyzer in sequence, which took approximate 3 min per cycle. Every 3-min measured CH_4_ value was expressed as grams per day as follows: CH_4_ (g) per measurement time interval/measurement interval (min) × 1,440 min in 24 h.

The Insentec feed bin system recorded entry and exit time and feed weight for each eating event during the day allowing the calculation of feeding time (min), intake (g), and intake rate (g/min) for each visit to the bin. However, during a meal, the animal sometimes takes the head out of the feed bin to chew and then goes back in, resulting in several consecutive recordings that are part of one meal. It was, therefore, necessary to define a meal criterion with start and end times. Here, we define meal criteria as described previously (Tolkamp et al., 2000; von Keyserlingk and Weary, 2010) based on the frequency distribution of intervals (feed bin exit time to next entry time) expressed on a log scale. The bimodal pattern was apparent with 20 min at the intersection between the 2 peaks. Therefore, for the current study, a 20-min interval was used as the threshold to define if a visit to the feed bin fell within a meal or if a new meal started. This interval for meal criteria was in a similar range of 17.9 to 29.8 min as previously found in growing heifers (DeVries and von Keyserlingk, 2009a,b).

Then, the start and end time of each meal was aligned with the respiration chamber data to identify the CH_4_ emissions measurements during each meal. The multiple CH_4_ values (which were already expressed as g/d as described above) within a meal were averaged to generate the CH_4_ production within a meal. The 24-h measured CH_4_ production (g/d) was calculated by averaging all ~3-min CH_4_ values.

Deming regression was performed to compare daily 24-h measured CH_4_ and daily CH_4_ calculated during mealtime (Linnet, 1993). Deming regression allows fitting a straight line to 2-dimensional data where both variables (X and Y) are measured with error. Bland-Altman (Bland and Altman, 1986) mean difference plot was generated to identify potential systematic bias and outliers in the data. Pearson correlations of feeding behavior parameters with 24-h CH_4_ production and yield were also performed. The data were analyzed using package ‘mcr' in R version 3.4.2 (R Core Team, 2018).

Average 24-h CH_4_ emissions for the 8 heifers were 319 ± 24.0 g/d compared with 313 ± 22.7 g/d when estimated from mealtime measurements (Table 1). The mealtime CH_4_ emissions were on average recorded during 13 meals/d, lasting 34.6 min/meal and 436 min/d (~7.3 h; Table 2). Mealtime CH_4_ had a strong correlation with 24-h measured CH_4_ production (r = 0.88) as determined using Deming regression (Figure 1). The 95% confidence interval of the slope between 24-h measured CH_4_ and mealtime CH_4_ estimated using Deming regression was 0.77 to 1.35, which indicates that the slope was not different from 1. There was no trend visible in the Bland-Altman plot, suggesting that there was no systematic bias in CH_4_ estimates based on simulated mealtime CH_4_ measurements.

**Table 1:** Methane production (g/d) and yield (g/kg DMI) estimated from measurements during mealtime at the feed bin and from 24 CH_4_ measurements in growing heifers fed ad libitum alfalfa silage in respiration chambers

Item	Mean	SD	Maximum	Minimum	CV
24-h CH_4_^1^ (g/d)	262	24.0	319	226	9.2
24-h CH_4_ (g/kg of DMI)	25.3	2.15	29.0	20.8	8.5
Mealtime CH_4_ (g/d)	276	22.7	313	241	8.2
Mealtime CH_4_ (g/kg of DMI)	25.8	3.07	35.3	20.8	11.9

1 The 24-h measured CH_4_ production was calculated by averaging all ~3-min CH_4_ values recorded in a 24-h period by the chamber system.

**Table 2:** Feed intake and feeding behavior parameters of 8 growing heifers fed ad libitum alfalfa silage and Pearson correlation (r) of feeding behavior parameters with daily methane production (CH_4_p) and yield (CH_4_y), all measured in respiration chambers

Item	Mean	SD	Maximum	Minimum	CV	Correlation (r) with:	
						CH_4_p^1^	CH_4_y
DMI (kg/d)	10.8	1.56	14.2	7.5	14.5	0.71*	−0.77*
Feed bin visit frequency (/d)	97	34	169	47	35.1	0.06	−0.45*
Number of meals^2^ (/d)	13	2.9	22	9	21.7	0.78*	−0.11
Eating time (min/meal)	14.7	3.74	22.3	7.8	25.4	−0.50*	0.13
Meal duration (min/meal)	34.7	13.4	71.4	19.4	38.8	−0.35	−0.16
Total eating time (min/d)	186	25.3	241	132	13.6	0.04	0.14
Total mealtime (min/d)	436	112.5	642	232	25.8	0.08	−0.29
Meal size (kg of DM/meal)	0.8	0.15	1.21	0.59	19.2	−0.25	−0.57*
Eating time eating rate (g of DM/min)	57.4	12.7	91.4	39.6	22.1	0.47*	−0.48*
Mealtime eating rate (g of DM/min)	30.4	8.87	46.7	17.2	29.2	0.30	−0.10
Interval between meals (min/interval)	75.6	16.02	110.8	40.3	21.2	−0.26	−0.24

1 The 24-h measured CH_4_ production was calculated by averaging all ~3-min CH_4_ values recorded in a 24-h period by the chamber system.

2 Meal criteria was defined as described previously (Tolkamp et al., 2000; von Keyserlingk and Weary, 2010) based on the frequency distribution of intervals of the feed bin exit time to next entry time expressed on a log scale (Figure 1).

* *P* < 0.05.

**Figure 1:**
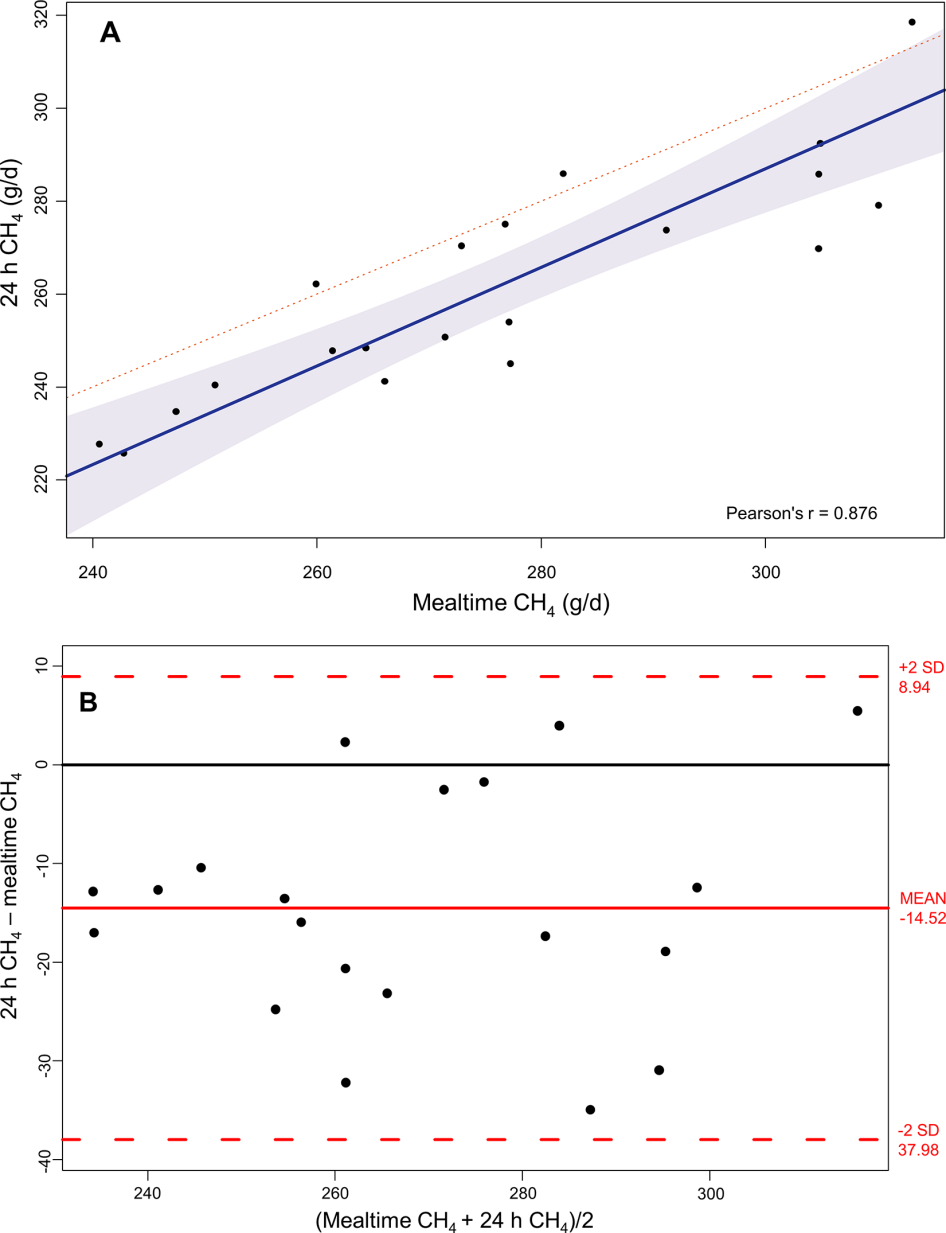
Deming regression (A) and Bland-Altman plot (mean difference plot; B) of CH_4_ production during mealtime at the feed bin (expressed as g/d) with 24-h measured CH_4_ production (g/d) by 8 growing heifers fed ad libitum alfalfa silage in respiration chambers. The shaded area indicates the 95% confidence interval; this was −111.5 to 48.9 for the intercept (−31.32) and 0.77 to 1.35 for the slope (1.06). The 24-h measured CH_4_ production was calculated by averaging all ~3-min CH_4_ values recorded in a 24-h period by the chamber system.

These results are consistent with the findings of Troy et al. (2016), who compared CH_4_ emissions determined with a custom-built hood system over Insentec feed-bins, with one open side to allow access to the feed by the animal, followed by measurements in respiration chambers with growing beef cattle in 2 experiments fed a range of diets (R^2^ = 0.64 in experiment 1 and R^2^ = 0.24 in experiment 2). However, absolute values were much lower (~3×) with the feed bin system than in respiration chambers and including diet fed in the multiple-regression model greatly improved the prediction of daily CH_4_ using feed bin CH_4_ data (concordance correlation from 0.55 increased to 0.79). In contrast, Derno et al. (2013) concluded that short-term CH_4_ measurements during feeding at the feed bin did not reflect average daily CH_4_ production based on respiration chamber data with dairy cows. However, this conclusion was based on time series analysis cross-correlation (correlation between 2 times series at lags) between feed intake and CH_4_ emissions, which is different from an analysis where CH_4_ measured during multiple feeding events in a day is averaged and then compared with 24-h measured CH_4_ emissions, as in the current study and the study of Troy et al. (2016).

It is not very useful to compare CH_4_ production estimates (g/d) to findings of other studies because DMI is the main driver of CH_4_ production (Charmley et al., 2016; Jonker et al., 2017); however, CH_4_ emissions per unit of DMI (yield) can be compared when animals are fed similar diets and feeding level. The CH_4_ yield in the current study averaged 25.3 g/kg DMI (range, 20.8–29.0 g/kg DMI; Table 1), which is in a similar range as for growing, dry, and lactating cattle fed forage-based diets (18.5–25.8 g/kg DMI; Jonker et al., 2020). Other studies measuring CH_4_ during all feeding events at the feed bin, using GreenFeed systems, also found CH_4_ yields in a similar range for growing heifers fed alfalfa cubes (20.7–22.7 g/kg DMI; Flay et al., 2019) and growing beef cattle fed concentrate-based diets (21.1–23.7 g/kg DMI; Biswas et al., 2018). These suggest that CH_4_ yields based on measurement to the feed bin can provide similar estimates to those measured during 24-h periods. However, the number of diets and animal measurements tested using this system are currently limited and the conclusion of Derno et al. (2013) suggested that mealtime CH_4_ emissions could not be used to estimate 24-h CH_4_ emissions. Therefore, further studies using other cattle categories and feeding different diets should be carried out to come to more robust conclusions about the accuracy of measuring CH_4_ during all feeding events only compared with 24-h measured emissions.

The heifers ate their feed on average in 13 meals/d, consuming 800 g of DM/meal, lasting 34.6 min/meal, and at a rate of 30.4 g of DM/min in the current study (Table 2), which was a similar range as previously found in growing dairy heifers who ate their feed in 6.8 to 11.1 meal/d, consuming 520 to 980 g/meal, lasting 26.0 to 62.9 min/meal, and at a rate of 37.7 to 57 g of DM/min (DeVries and von Keyserlingk, 2009a,b). Feeding behavior parameters that were correlated with CH_4_ production in the current study were a positive correlation (r = 0.78) with the number of meals per day and a negative correlation (r = −0.50) with average eating time per meal (min/meal). Previously, intake time was found to correlate (concordance correlation) positively with CH_4_ production in 2 studies (Muñoz-Tamayo et al., 2019; Ramirez-Agudelo et al., 2019). In the current study, total daily mealtime and total daily eating time had very weak correlations with CH_4_ production. There was also only a very weak correlation of daily DMI with daily mealtime and daily eating time (data not shown), which likely explains why intake time was a poor predictor of CH_4_ production in the current study.

Feeding behavior parameters that were correlated with CH_4_ yield in the current study were a negative correlation with the number of visits to the feed bin (r = −0.45), average meal size (r = −0.57), and average daily eating rate (r = −0.48). Llonch et al. (2018) also found a negative association between the number of visits to the feed bin and CH_4_ yield in growing beef cattle, supporting the finding of the current study. Offering less frequent and larger meals to lactating dairy cows and sheep was previously found to result in lower CH_4_ yield (Müller et al., 1980; Swainson et al., 2011), suggesting that the relationships of feeding behavior with CH_4_ yield in the current study make sense from a biological point of view.

In summary, CH_4_ measured during meals was similar to 24-h measured CH_4_ output in growing heifers fed ad libitum alfalfa silage in respiration chambers. Some feeding behavior parameters, based on feed bin visits, explained some of the variation in CH_4_ production and yield.
